# On the Importance of Pnictogen and Chalcogen Bonding Interactions in Supramolecular Catalysis

**DOI:** 10.3390/ijms222212550

**Published:** 2021-11-21

**Authors:** Antonio Frontera, Antonio Bauza

**Affiliations:** Departament de Química, Universitat de les Illes Balears, Crta. de Valldemossa km 7.5, 07122 Palma de Mallorca, Baleares, Spain; toni.frontera@uib.es

**Keywords:** σ-hole interactions, pnictogen bonding, chalcogen bonding, supramolecular chemistry, catalysis

## Abstract

In this review, several examples of the application of pnictogen (Pn) (group 15) and chalcogen (Ch) bonding (group 16) interactions in organocatalytic processes are gathered, backed up with Molecular Electrostatic Potential surfaces of model systems. Despite the fact that the use of catalysts based on pnictogen and chalcogen bonding interactions is taking its first steps, it should be considered and used by the scientific community as a novel, promising tool in the field of organocatalysis.

## 1. Introduction

Elements belonging to groups 13–18 and covalently bound to electron withdrawing groups (EWG) possess an inherent ability to interact with Lewis bases, such as lone pair donors, π-systems and anions [1,2,3,4,5,6,7,8,9,10,11,12,13]. Since the first use of the electropositive site to describe the hydrogen bonding (HB) interaction, it became commonplace to use the name of the group to which the electrophilic atom belongs to name noncovalent interactions (NCI) involving electrophilic and nucleophilic sites [14,15]. In this context, the International Union of Pure and Applied Chemistry (IUPAC) has already recommended the terms halogen bond (HaB) [16] and chalcogen bond (ChB) [17] for naming the NCIs involving atoms from groups 17 and 16, respectively. Furthermore, aerogen or noble gas bonding (NgB, group 18) [12], pnictogen bonding (PnB, group 15), [18,19] tetrel bonding (TtB, group 14) [20], and triel bonding (TrB, group 13) [7] have been used for main group elements not yet recommended by the IUPAC. In addition, matere bonding (MaB, group 7) [21], osme bonding (OmB, group 8) [22], spodium bonding (SpB, group 12) [23] and regium or coinage bonding (CiB, group 11) [24], have been recently used to refer to NCIs where elements from groups 7, 8, 11 and 12 act as Lewis acids, and to differentiate them from classical coordination bonds [24,25,26,27]. Many studies have reported the feasibility of σ-hole interactions as an alternative to HB interactions in a broad spectrum of fields, such as host–guest chemistry, catalysis, supramolecular chemistry, membrane transport, crystal engineering, etc., [28,29,30,31,32,33,34,35]. A great deal of review articles and book chapters are available in the literature describing the basic features and potential applications of this group of NCIs [28,29,30,31,32,33,34,35,36,37,38,39,40,41,42,43,44,45]. Moreover, several studies have been devoted to the comparison of σ-hole interactions to the HB in terms of energetic and geometric features [46,47,48,49,50,51].

**Figure 1:**
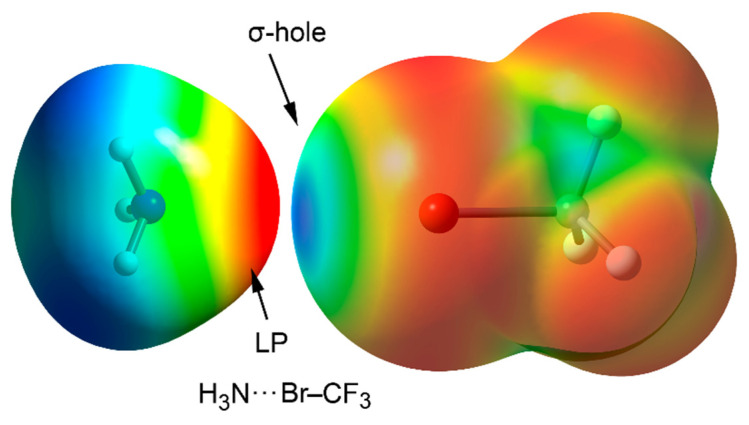
MEP surface of an σ-hole complex between NH_3_ and BrCF_3_ molecules acting as electron donor and acceptor moieties, respectively. Electropositive regions are represented in blue and electronegative in red.

The attractive interaction (EWG–X···A) between a p-block element (Lewis acid, X) and a σ-hole acceptor (A, Lewis base, anion or π-system) is supported by the sum of different contributions (see Figure 1 for a Molecular Electrostatic Potential (MEP) surface of a prototypical CF_3_Br···NH_3_ complex). These correspond to electrostatics, charge transfer, orbital mixing, polarization and dispersion forces [52,53]. Firstly, electrostatics is understood as the attraction between the electron-rich region (belonging to the electron donor (A)) and a region of diminished electrostatic potential located at the prolongation of the EWG–X bond, known as the σ-hole (which belongs to the electron acceptor). Secondly, dispersion and polarization terms are also important contributors to those NCIs involving the heavier elements of the groups, which are easily polarized [45,54,55,56,57,58,59,60]. Lastly, the orbital term is rationalized as the global stabilization of the complex owing to the electron donation from the electron-rich atom or group of atoms (e.g., LP(A) or π(A)) to an antibonding EWG–X orbital (σ * (EWG–Y). Since the polarizability of the atoms increases when descending the groups of the periodic table, the electropositive region of the σ-hole increases if the EWG–X bond is more polarized, thus resulting in a reinforcement of the NCI. In addition, increasing the electron-withdrawing ability of EWG is another way to polarize the EWG–X bond. Thus, the combination of heavy elements and strong EWGs increases the positive potential and size the σ-hole, which results in the strengthening of the NCI. This has led to an increase of the number of investigations [61,62,63,64,65,66,67,68,69,70,71,72,73,74] involving elements of groups 14-18, which has paved the way of new lines of research [14,15].

## 2. Results and Discussion

### 2.1. Pnictogen Bonding

A great deal of studies dealing with PnBs are already available in the literature [43,44,75,76,77,78,79,80,81,82,83,84,85]. However, those presenting experimental evidence of the interaction are scarce [86,87,88,89].

**Figure 2:**
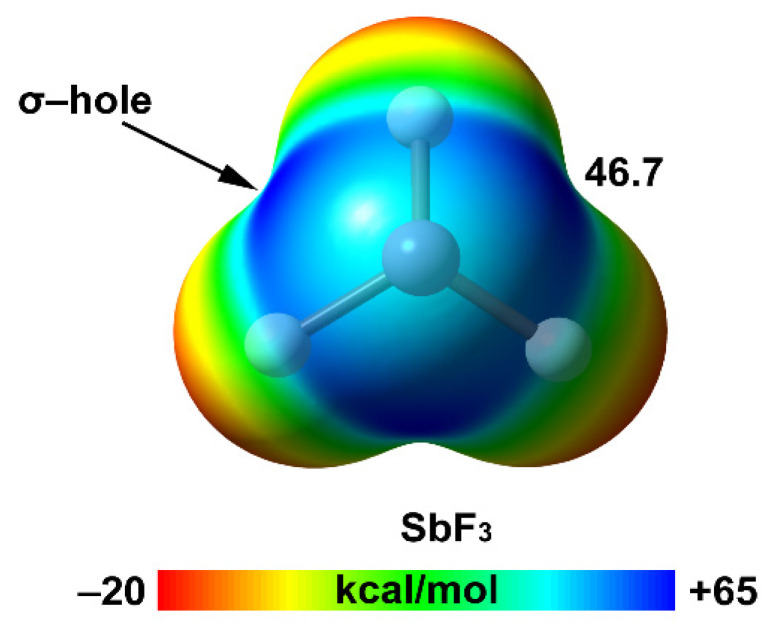
MEP surface of SbF_3_ at the MP2/def2-TZVP level of theory at the 0.001 a.u. isovalue (energy in kcal/mol).

Table 1 gathers the atomic polarizability values (α) and van der Waals radii of the pnictogen elements from period two to five. The atomic α value increases from 5.3 a.u. in N to 30.8 a.u. in Sb. As noted, the difference in α between N and P is substantial (~3x), and small between P and As. Lastly, the difference is again relevant between As and Sb. In Table 1, the MEP values at the σ-holes of the fluoride derivatives of the four Pn elements are also gathered, while Figure 2 includes the MEP surface for SbF_3_. As noted, the MEP values at the σ-hole become more positive when descending in the group, similarly to the well-characterized halogen bond interaction.

Figure 2 shows the MEP surface of SbF_3_ as an illustrative example of the pnictogen group. As noted, it presents an ample region of positive MEP values where the global maximum is located approximately in the opposite direction to the Sb–F bonds. In addition, the large and negative MEP values at the F-atoms reveal a strong polarization of the Sb–F bonds.

#### Arsenic and Antimony

Arsenic and antimony are known to be involved in stronger PnBs than N and P, as evidenced by the MEP analysis shown above. Their use as efficient anion binders [90] has been recently reported, with antimony being the most promising Pn atom to be used for anion recognition. In another study, Gabbaï and collaborators have reported bis-stilbonium as a supramolecular synthon to build a preorganized host [91] involved in several PnBs interactions with electron-rich species. Additionally, Cozzolino and coworkers have synthesized anion receptors based on bidentate anion-binding motifs containing two Sb(III) centers, bridged by either oxygen or sulfur atoms acting as PnB donors [92].

In the field of organocatalysis, the number of studies devoted to the development of new functional PnB-based catalysts has rapidly increased during recent years [93,94,95,96,97,98,99,100,101,102]. In this section, several selected examples involving PnB-based catalysts are discussed. For each example an MEP surface of the Pn catalyst is shown, in addition to a plausible transition state/reaction intermediate stabilization scheme.

Benz and collaborators [93] compared the catalytic activity of a series of Pn catalysts using P, As and Sb to their Ch (Se and Te) and Ha (Br and I) analogues. More precisely, the authors synthesized a series of phenyl and pentafluorophenyl (PFP)-substituted Pn, Ch and Ha moieties (see Table 2), and evaluated the dissociation constants (K_D_ values) of their corresponding chloride complexes by ^19^F NMR spectroscopic titration with TBACl in THF.

**Figure 3:**
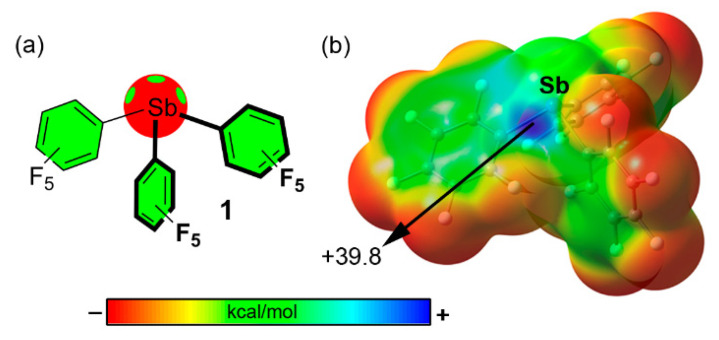
(**a**) Schematic representation and (**b**) MEP surface (0.001 a.u.) of compound **1** (B3LYP/def2-TZVP level of theory) [93]. All MEP surfaces corresponding to Pn and Ch catalysts have been performed using the Gaussian16 calculation package [106].

Among them, Sb(PFP)_3_ (compound **1**), gathered in Figure 3a, achieved the best results in terms of dissociation constant (K_D_) of the chloride complex (19 ± 7 μM). As a likely explanation, the authors recalled the presence of three accessible σ-holes on the catalyst surface (only one is shown in Figure 3b) which exhibited MEP values of +38.9 kcal/mol.

**Figure 4:**
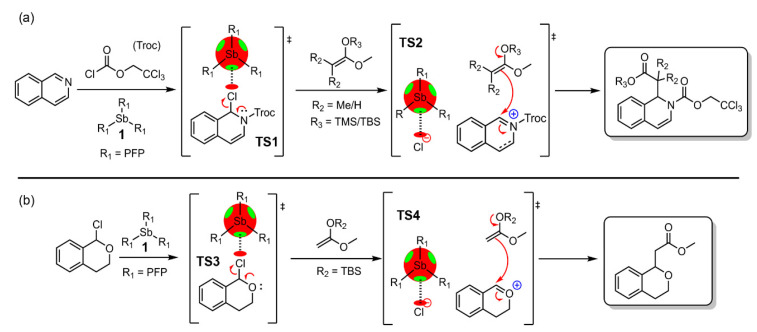
(**a**) Proposed reaction pathways of the Reissert-type substitution of isoquinolines and (**b**) the transformation of 1-chloroisochromane to an ester, including a schematic representation of the plausible stabilized transition states (TS1-4) through Sb-PnBs.

Bearing these promising results in mind, Benz and collaborators successfully applied PnB catalysis to two organic reactions. The first was the Reissert-type substitution of isoquinoline, which is known to be catalyzed by different anion-binding catalysts [103,104,105]. As shown in Figure 4a, compound **1** accelerates the reaction by stabilizing the rate-limiting transition state 1(TS1), that is, the elimination of chloride following the addition of Troc chloride. In a posterior stage, the resulting cationic intermediate reacts with the nucleophile to afford products (TS2). Interestingly, after an initial screen of solvents and conditions, the authors obtained a 51% of yield within 30 min at −100 °C in THF using 5 mol% of catalyst.

The authors also achieved success in catalyzing the more challenging transformation of 1-chloroisochromane to an ester, a reaction that proceeds through chloride abstraction in TS3 and successive attack of a silyl enol ether (see Figure 4b). The authors obtained a 91% yield after 55 h at −78 °C in THF using a 20 mol% of compound **1**. In this case, both TS3 and 4 are also stabilized by Sb···Cl PnBs, which is similar to the Reissert-type substitution of isoquinolines, resulting in the corresponding ester.

**Figure 5:**
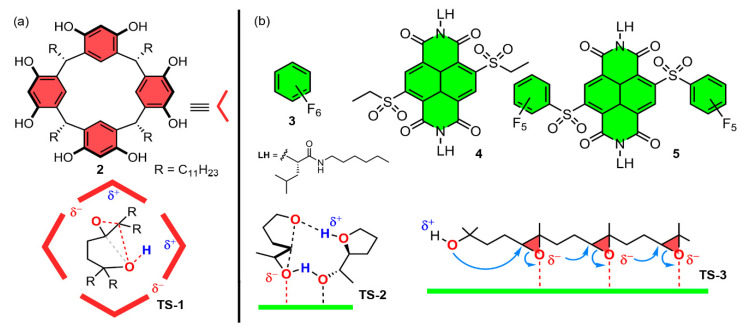
Schematic representation of several catalysts including (**a**) π-basic and (**b**) π-acid surfaces. The plausible transition states including cation–π interactions in confined space of π-basic capsules (TS-1) and anion–π interactions in the cascade cyclization of a tetraepoxide (TS-2 and TS-3) are also included.

Additionally, Matile’s group has carried out several studies on ether cyclization reactions [94,95,96,98]. In their study [96], Hao and collaborators carried out a comparative analysis of the use of electron-rich and electron-poor π-surfaces and PnB-based catalysts (see Figure 5 for some of the catalysts used) on bioinspired ether cyclization reactions. More precisely, the authors explored four epoxide-opening ether cyclizations as standards for the comparative assessment of catalytic systems.

**Figure 6:**
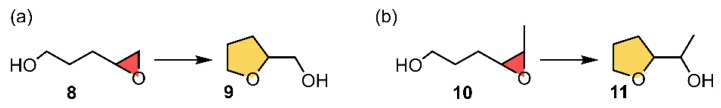
Schematic representation of two epoxide-opening ether cyclizations studied by Hao and collaborators [94] yielding compounds (**a**) **9** and (**b**) **11**.

Firstly, the authors studied the ring-opening reaction of two ether derivatives (**8** and **10**) to yield products **9** and **11**. In the first transformation (Figure 6a), the Baldwin-compatible 5-exo-tet oxolane product **9** was obtained using both capsule **2** and compound **3** as catalysts. Although the initial results were not promising, since for instance the conversion using hexafluorobenzene (**3**) as a solvent catalyst reached around 10% in 7 h, the authors incremented the concentration of product **9** from the beginning of the reaction while using catalyst **2**, thus accelerating the rate of cyclization up to k_cat_/k_cat(0)_ = 30 ± 10.

In a parallel way to substrate **8**, the cyclization of the 4,5-epoxy alcohol **10** with an extra methyl in the *cis* position on the epoxide solely yielded the Baldwin product **11** (Figure 6b). In this case, the reaction within capsule **2** was faster than that for the previous reaction (Figure 6a). Furthermore, the addition of extra product **11** at the beginning slowed down rather than accelerated the reaction, which was consistent with mildly competitive product encapsulation. On the other hand, the use of catalyst **3** achieved a faster conversion to product **11** and showed strongly autocatalytic behavior. In sharp contrast to the previous reaction, the addition of product **11** at the beginning of the cyclization of **10** using **3** did not accelerate conversion. However, the replacement of hexafluorobenzene solvent catalyst **3** by catalyst **4**, which exhibited a larger π acidity (an electron-poor π system), showed noticeable autocatalysis with **10**. In Figure 5a,b, a schematic representation of the plausible transition states is shown (TS-1 and TS-2), involving the “accommodation” of the substrate within capsule **2** (TS-1) and the formation of two hydrogen bonds (one hydrogen-bond acceptor and one hydrogen-bond donor) in the activation of both nucleophile and leaving group in TS-2.

**Figure 7:**
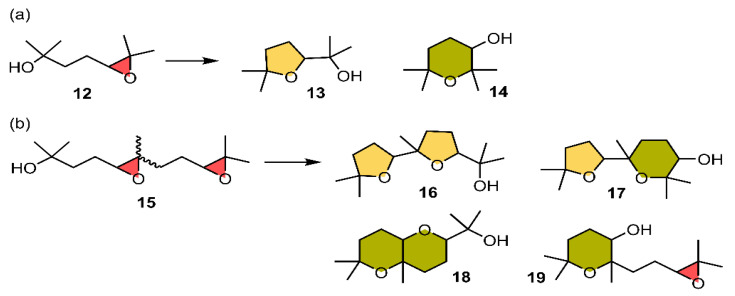
Schematic representation of two epoxide-opening ether cyclizations studied by Hao and collaborators [94] yielding compounds (**a**) **14** and (**b**) **16** to **19**.

**Figure 8:**
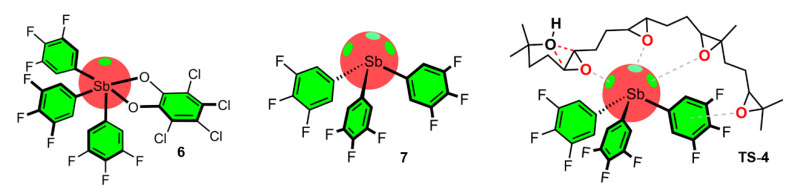
Schematic representation of several catalysts including multiple pnictogen bonding for cascade cyclization of a tetraepoxide on Sb(III) catalyst [94] (TS-4).

The authors also analyzed the cyclization of the 4,5-epoxy alcohol **12** with four extra methyl groups around the nucleophile and electrophile to yield the Baldwin oxolane product **13** together with the anti-Baldwin oxane product **14** (Figure 7a). In capsule **2**, the reaction further accelerated, likely due to stabilization by cation–π interactions (TS-1, Figure 5a). On the other hand, the use of hexafluorobenzene **3** revealed a similar result as in the case of compound **8**. In this scenario, the addition of product **13** at the beginning of the reaction of **12** in **3** accelerated the reaction noticeably, although not enough to surpass the results obtained using supramolecular capsule **2**, which performed better than the HB and PnB catalysts used (Figure 8). The latter accelerated the reaction though the establishment of multiple PnBs with the three available σ-holes present in the Sb atom and a lone pair-π interaction involving the fluorinated ring moiety (TS-4).

In the last stage of their study, diepoxide **15** was used, and the reaction followed ^1^H NMR spectra (Figure 7b). In this case, **15** was initially used as a *cis*/*trans* mixture of isomers with a 6:4 ratio. The product mixtures contained the oxolane dimer **16** resulting from a cascade cyclization following Baldwin rules, the oxolane-oxane dimer **17** resulting from a Baldwin cyclization followed by an anti-Baldwin cyclization, and the fused bicycles **18** and **19** produced by an anti-Baldwin cyclization followed by a Baldwin and an anti-Baldwin cyclization, respectively. Interestingly, the reaction carried out within capsule **2** afforded **17** as the main product, with high apparent diastereoselectivity, with only the one **16** diastereomer as a minor side product. The formation of **18** and **19**, both produced from an initial anti-Baldwin cyclization, was almost completely suppressed. This diastereoselective formation of isomers of **17** could possibly be explained by the ability to fold into the most globular structure, as outlined in TS-1.

**Figure 9:**
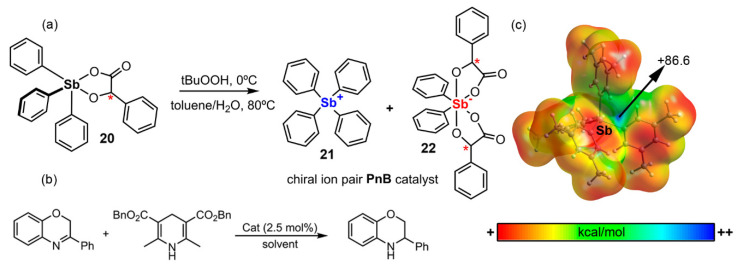
(**a**) Schematic representation of the synthetic procedure to obtain the chiral ion pair PnB catalyst (the methyl groups bound to the benzene rings have been omitted for sake of clarity), (**b**) reaction scheme of the benzoxanine reduction with Hantzch ester and (**c**) MEP surface of compound **21** (B3LYP/def2-TZVP). The energy value shown at the selected point in the surface is given in kcal/mol (0.001 a.u.). Chiral centers are denoted by a red * in the figure.

Zhang and collaborators [97] developed a new family of chiral PnB-based catalysts (see Figure 9a). Concretely, **20** was obtained through the reaction of triarylstibine and mandelic acid under oxidative conditions and used as a catalyst in the reduction of benzoxazine with Hantzch ester (Figure 9b). After purification, the authors observed a decrease in the catalytic performance of **20** without noticing a clear decrease in enantioselectivity, as well as an unusual rate acceleration at the middle stage of the reaction. After further investigation, the authors assigned the catalytic active specie to the chiral antimony (V) cation/anion pair (compounds **21** and **22** in Figure 9a). As noticed in Figure 9c, the MEP surface analysis of **21** reveals an accessible and very positive Sb σ-hole (+86.6 kcal/mol), which makes it a very strong PnB donor. This ion pair promoted the reduction of benzoxazine efficiently in 2 h, obtaining the product with 99% yield with 80% ee.

Consequently, compound **20** analogues with different mandelic acid ligands were prepared and tested in different reaction conditions. On the other side, different substituents were used in benzoxazine to assess their influence on the reaction global performance. Lastly, to gain mechanistic insights into the chiral antimony (V) cation/anion pair catalyzed asymmetric transfer hydrogenation, nonlinear effect studies were conducted. The authors observed a weak negative nonlinear relationship between ee of the catalyst and ee of the product. This phenomenon indicated the presence of ligand exchange and/or catalyst aggregation during the course of the reaction, which was further confirmed by ^1^H NMR spectra [97].

**Figure 10:**
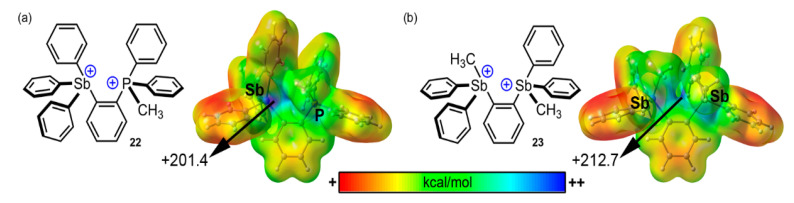
(**a**) Schematic representation of compounds **22** and **23** and (**b**) their corresponding MEP surfaces. Energies at the selected points of the surface are given in kcal/mol (0.035 a.u.).

The last selected example of PnB catalysis encompasses the study from Yang and coworkers [98] where they synthesized monodentate and bidentate phosphonium-stilbonium and bis-stilbonium cations as PnB catalysts. More precisely, bidentate Pn compounds **22** and **23** were synthesized and X-ray characterized. Both of them are dications, therefore bearing either one Sb(III) and one P(III) or two Sb(III) in their structure (see Figure 10). As noticed, their corresponding MEP surfaces exhibit very positive Sb and P σ-holes. In the case of compound **22**, only the Sb σ-hole was accessible with an MEP value of +201.4 kcal/mol, whilst in compound **23** both Sb σ-holes presented spatial accessibility and an MEP value of +212.7 kcal/mol (B3LYP/def2-TZVP level of theory).

In a similar way to the study of Zhang and collaborators [97], Yang et al. used compounds **22** and **23** as catalysts for the transfer hydrogenation reaction between Hantzch ester and quinolines (see Figure 9b,10). Furthermore, the authors also synthesized monostilbonium cations, which presented lower catalytic activity compared to bidentated Pn catalysts **22** and **23**. Particularly, the use of **22** afforded a conversion of 90% after 1 h, which was assigned to the presence of two adjacent cationic Sb centers which can actuate in a cooperative way to activate the substrate.

**Figure 11:**
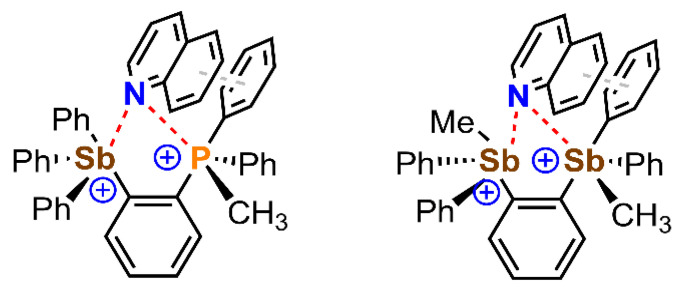
Schematic representation of the plausible transition state stabilization through bifurcated PnB and π–π stacking interactions involving compounds **22** and **23**.

Finally, DFT calculations were used to simulate the double activation of the substrate by the dicationic systems, a step that would precede transfer of a hydride from the Hantzsch ester. Using a simplified computational model, the authors predict the lowest energy binding mode between the substrate and the catalyst (see Figure 11). As noted, for both catalysts **22** and **23,** a bifurcated N···Sb/P PnB was proposed in addition to a stacking interaction involving the π–systems of the quinoline and a phenyl ring bound to one of the Pn atoms, which further assisted in the stabilization of the supramolecular complex.

### 2.2. Chalcogen Bonding

Among chalcogens, organotellurium compounds such as tellurophene and bis(tellurophene) derivatives are gaining interest in supramolecular catalysis and anion recognition phenomena [11,107,108,109,110,111].

**Figure 12:**
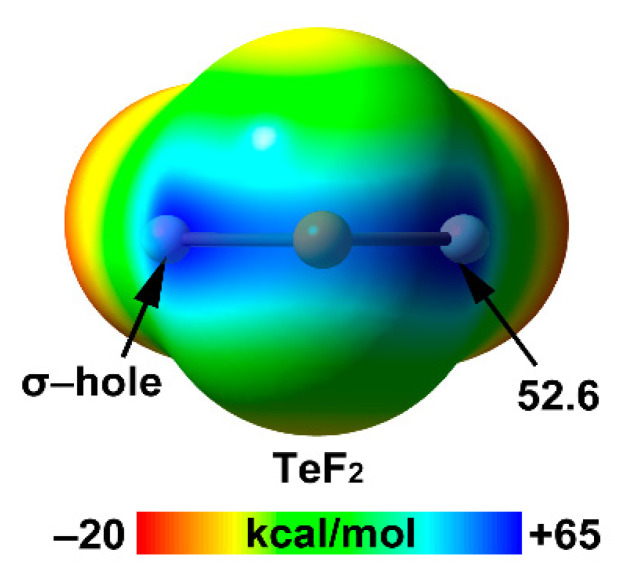
MEP surface of TeF_2_ using the 0.001 a.u. isosurface at the MP2/def2-TZVP level of theory (energy in kcal/mol).

In Table 3, the atomic polarizabilities (α) and van der Waals radii of the chalcogen elements from period two to five are displayed. As noted, the atomic α value increases from 3.0 a.u. in O to 25.9 a.u. in Te. Interestingly, the difference in the atomic polarizability between O and S is quite large (~4x), which is in line with the behavior observed in Pn atoms. On the contrary, the differences between S and Se or Se and Te are negligible. The MEP values at the σ-holes of the fluoride derivatives of the four Ch elements studied in this section are also given in Table 3, and the MEP for TeF_2_ molecule is represented in Figure 12. They show the expected trend with σ-hole values increasing from 16.8 kcal/mol in OF_2_ to 52.6 kcal/mol in TeF_2_.

Figure 12 shows the MEP surfaces of TeF_2_ as an illustrative example of the whole series. As noted, TeF_2_ exhibits an extended region of positive MEP values in the molecular plane, with two σ-holes located approximately on the extension of the F–Te bonds. A similar behavior is observed in SbF_3_ (see Figure 2).

#### Selenium and Tellurium

Several studies available in the literature are focused on direct applications of ChB in catalysis [112,113,114,115,116,117,118,119,120,121,122,123,124,125,126], showing interesting and clever ways of the use of ChBs to stabilize negatively charged transition states.

**Figure 13:**
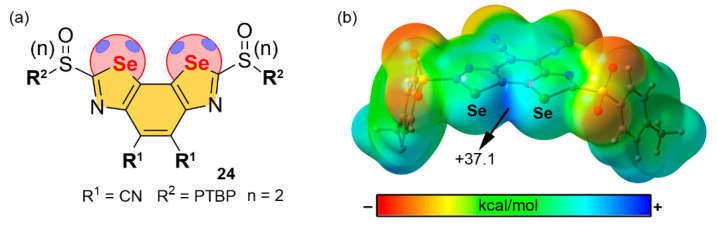
(**a**) Schematic representation of compound **24** (PTBP stands for *para*-tercbutylphenyl) and (**b**) its MEP surface at the B3LYP/def2-TZVP level of theory. The energy value at the selected point of the surface is given in kcal/mol (0.001 a.u.).

In 2017, Benz and collaborators [112] proposed neutral benzodiselenazole (BDS) as a prototypical molecular synthon for the catalysis of the transfer hydrogenation of quinolines (see Figure 13). Concretely, the authors proposed several functionalized BDS as structural motifs involving Se ChB donors using zero, one or two sulfoxide moieties and the substituents R^1^ and R^2^, which accounted for CN, CH_3_ and iBu groups (R^1^) and *para*-tercbutylphenyl (PTBP), adamantane and octyl groups (R^2^) (see Figure 13a). In this regard, the MEP analysis of one of the most promising candidates (**24**, see Figure 13b), as the ChB donor is shown. As noticed, two strong Se σ-holes are present, located along the C–Se bonds and overlapping between the Se atoms (+31.7 kcal/mol).

**Figure 14:**
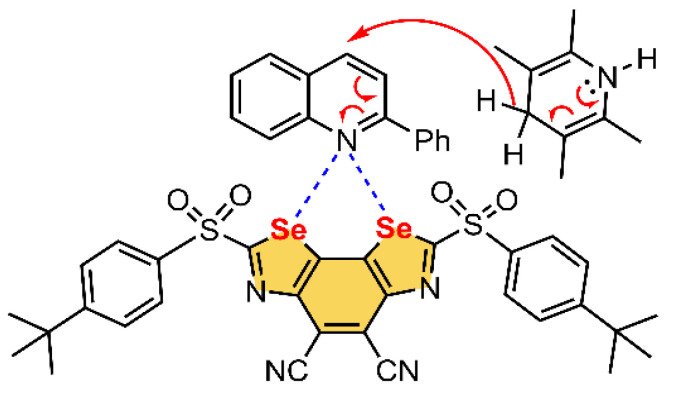
Schematic representation of the expected transition-state stabilization by a bifurcated Se ChB, involving compound **24**.

Using the ChB-donor ability of **24**, the authors demonstrated its catalytic activity in the hydrogenation reaction of quinolines (see Figure 14) through a transition-state stabilization in the focal point of the Se σ-holes of the BDS. Concretely, in the transition state, the negative charge carried out by the hydride is located on the endocyclic nitrogen, which can be stabilized through the formation of a bifurcated Se ChB, therefore resulting in rate enhancement of the transfer hydrogenation. The authors reported a catalytic rate enhancement of five orders of magnitude by compound **24**, with presented maximized σ-holes by two strong sulfone- and two strong cyano-acceptors.

**Figure 15:**
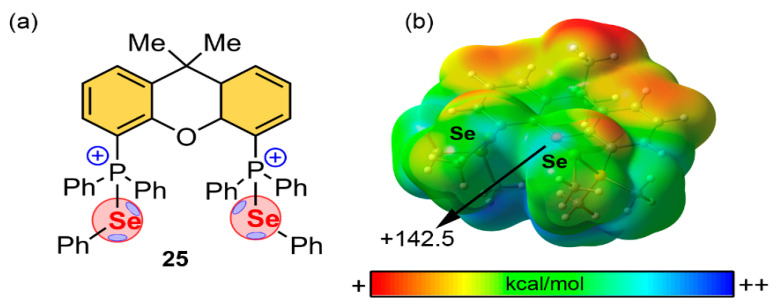
(**a**) Schematic representation of compound **25** and (**b**) its MEP surface at the B3LYP/def2-TZVP level of theory (Ph groups have been replaced by Me groups for sake of clarity). Energy value in kcal/mol (0.001 a.u.).

Other interesting examples are the studies by Wang [113,114] and Bao [115] and collaborators, which effectively designed a series of catalysts based on Ch···Ch interactions. In one of their works [114], the authors carried out the synthesis, X-ray and NMR characterization and the evaluation of the catalytic activity of a series of ChB catalysts for the Rauhut–Currier reaction based on dual Se···O ChBs (see Figure 15 for one of the most promising candidates). As noticed, there are two available Se σ-holes on the catalyst which exhibit an electrostatic potential value of +142.5 kcal/mol, owing to the positively charged nature of the molecule.

**Figure 16:**
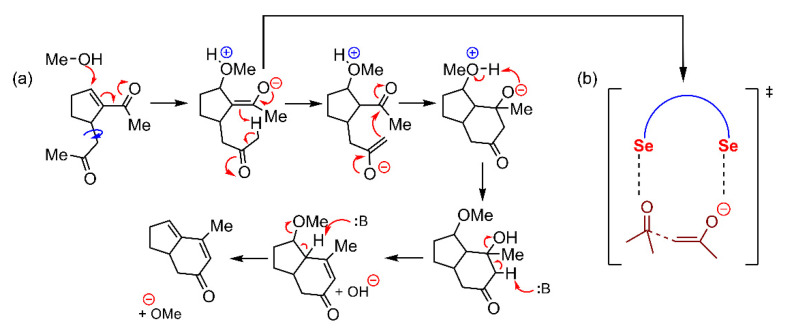
(**a**) Proposed reaction pathway of the Rauhut–Currier reaction and (**b**) Schematic representation of the plausible stabilized transition state through a dual ChB interaction.

The dual binding ability of this family of catalysts was used to interact with the two nucleophile species needed for the Rauhut–Currier reaction, which are an enone and an alcohol. Particularly, the simultaneous interaction of a ChB catalyst with these two reactants provoked a shift from the intermolecular alcohol addition of enone to an “intramolecular” manner, thus facilitating the addition process (see Figure 16 for the proposed reaction mechanism and transition state stabilization). To verify this hypothesis, the authors performed several experiments, altering the concentrations of either of the nucleophiles or modifying the substituents of the Se atom to evaluate the effect of the steric hindrance of the catalytic activity. They concluded that a proper balance between the concentrations of the two electron donors is essential, as the catalyst needs to simultaneously interact with both the alcohol and the enone to drive this reaction.

**Figure 17:**
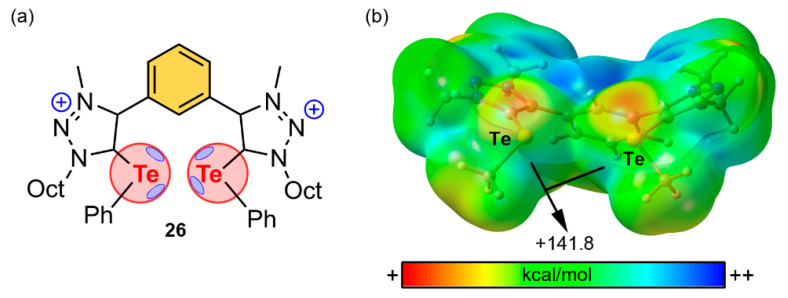
(**a**) Schematic view of compound **26** and (**b**) its MEP surface analysis at the B3LYP/def2-TZVP level of theory (Ph and Oct groups have been replaced by Me groups for sake of clarity). Energy value in kcal/mol (0.001 a.u.).

In 2019, Wonner and collaborators [116] proposed the synthesis and catalytic activity of a series of Ch-based organocatalyts. Figure 17 shows the chemical structure and MEP surface of compound **26**, a tellurium-based catalyst which exhibited a promising activity for the nitro Michael reaction. As noted in Figure 17b, the MEP surface of this compound shows two very positive electrostatic potential regions corresponding to the location of two Se σ-holes (+141.8 kcal/mol). Similarly, in the previous study from Wang and collaborators [113], the cationic nature of the catalyst provokes and enhancement of the σ-hole donor ability of the Te atoms. The authors achieved better reaction yields when using low coordinating counterions (such as BF_4_ or BARF), suggesting a higher accessibility of the Te σ-holes.

**Figure 18:**
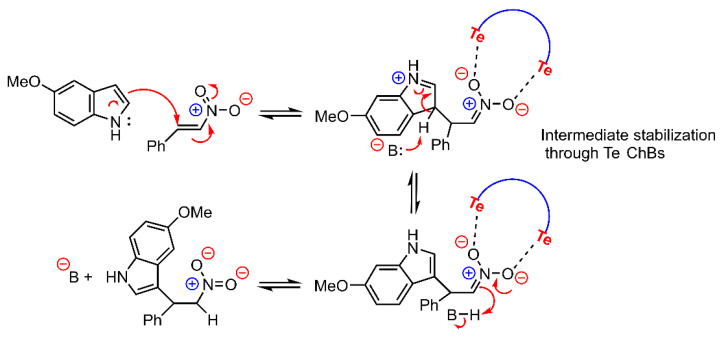
Schematic representation of the proposed reaction pathway for the nitro Michael reaction catalyzed by compound **26** with a plausible stabilization of the reaction intermediate through two simultaneous ChBs.

The proposed mechanism of action is shown in Figure 18, and as noted, compound **26** undergoes two simultaneous ChBs with the two negatively charged O atoms from the nitro derivative, thus likely stabilizing the reaction intermediate. This aspect was further demonstrated by DFT calculations (M062X-D3/def2-TZVP level of theory) which proposed Te···O distances of 2.49 and 2.53 Å and C–Te···O angles of 166 and 168°, in line with two directional ChB interactions.

**Figure 19:**
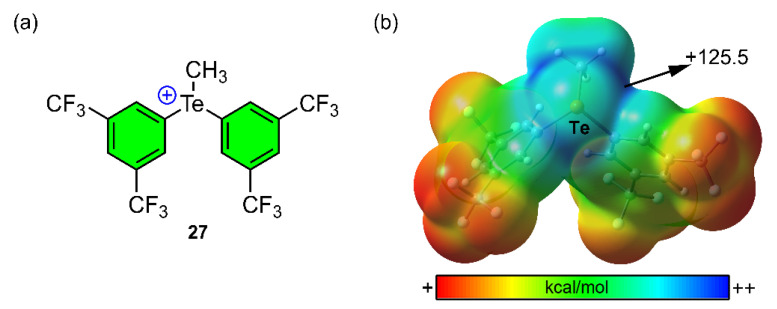
(**a**) Schematic view of compound **27** and (**b**) MEP surface of the catalyst at the B3LYP/def2-TZVP level of theory. Energy value in kcal/mol (0.001 a.u.).

As the final example, Weiss and collaborators [117] designed and synthesized a Tellurium organocatalyst (compound **27**, Figure 19a), able to efficiently catalyze three different reactions: the Friedel–Crafts bromination of anisole, the bromolactonization of ω-unsaturated carboxylic acids and the aza-Diels–Alder reaction between Danisehfsky’s diene and imines. In Figure 19b the MEP surface of compound **27** is shown, which involves CF_3_ substitution in both benzene rings. As noted, the MEP value around the three available Te σ-holes is very positive (+125.5 kcal/mol), which makes it suitable for the establishment of strong Te ChBs.

**Figure 20:**
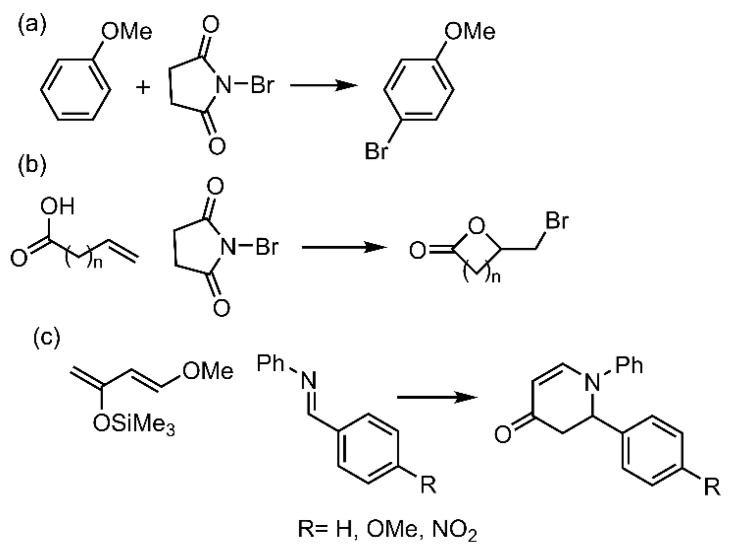
Schematic view of the three benchmark reactions used to evaluate the efficiency of the Te organocatalysts. (**a**) Friedel–Crafts bromination of anisole. (**b**) Bromolactonization of ω-unsaturated carboxylic acids. (**c**) Aza-Diels–Alder reaction between Danisehfsky’s diene and imines.

The authors used the three reactions shown in Figure 20 as benchmark tests to evaluate the effectiveness of Te catalyst **27**. Concretely, in the Friedel–Crafts bromination of anisole (Figure 20a), the authors used two aromatic substituents (–OMe and –CF_3_), obtaining very high yields in all the cases (80–90%). However, those catalysts using [BArF_4_]^−^ as a counterion achieve the best results, in agreement with previous studies [114], likely due to a greater Te σ-hole availability. They concluded that increasing or decreasing the catalyst amount did not affect the catalyst efficiency, and interestingly, even 0.5 mol% of catalyst still promoted this reaction very efficiently within 5 min. Finally, the authors also confirmed the catalyst stability by performing NMR monitoring of the reaction.

The second benchmark reaction used was the bromolactonization of *ω*-unsaturated carboxylic acids (Figure 20b). Concretely, a series of ω-unsaturated carboxylic acids was submitted to NBS in the presence of telluronium catalyst shown in Figure 20a. Interestingly, the expected bromolactones were obtained within 30 min in good quantitative yields for 4-to-6-membered rings, although in lower yields for larger rings, while no reaction occurred without the catalyst. The authors also confirmed the stability of the catalyst during the reaction. Finally, the authors further tested compound **27** on the aza-Diels–Alder-type reaction, using the Danishefsky’s diene condensation with various imines as a prototypical reaction. In this case, all the examined imines readily afforded the expected N-phenyl 2,3-dihydro-4-pyridinones in good-to-high yields with only 1 mol% of catalyst within 2 h at room temperature.

## 3. Conclusions

The data of MEP and polarizability data summarized in this review show that the polarizability of the p-block elements increases when moving from period two to five, with large differences between periods two and three. Moreover, the MEP values increase when descending in the group, similarly to the behavior observed for the polarizability. The selected examples shown in Section 2.1 and Section 2.2 of this review, which is not intended to be comprehensive, give significant experimental support to the fact that the elements from groups 15 and 16 have a strong tendency to establish directional PnB and ChB interactions with Lewis bases or anions. This in turn can be used for the stabilization of reaction intermediates as well as transition states, thus accelerating and improving reaction rates. Although the applications of these novel NCIs in this field of research are in its mere naissance, the use of catalysts based on PnB and ChB interactions should be considered and used by the scientific community as a novel and efficient tool in the field of organocatalysis.

## Figures and Tables

**Table 1:** Atomic polarizabilities (α, a.u.) of pnictogen (Pn) elements, van der Waals radii (R_vdW_, Å) and σ-hole MEP value (MEP, kcal/mol) of their trifluoride derivatives. Data extracted from ref. [63].

Pn	α	R_vdW_	MEP (PnF_3_)
N	5.3	1.55	15.9
P	16.9	1.80	27.4
As	21.6	1.85	38.5
Sb	30.8	2.06	46.7

**Table 2:** Catalyst candidates used and dissociation constants (K_D_ in μM) of chloride complexes in THF. Ph and PFP stand for phenyl and pentafluorophenyl moieties, respectively.

**Pn-Catalyst**	**K_D_**				
P(PFP)_3_	N/A				
As(PFP)_3_	13,300 ± 800				
Sb(Ph)_3_	N/A				
Sb(Ph)_2_PFP	N/A	Ch-Catalyst	K_D_	Ha-Catalyst	K_D_
SbPh(PFP)_2_	570 ± 70	Se(PFP)_2_	27,000 ± 4000	BrPFP	N/A
Sb(PFP)_3_	19 ± 7	Te(PFP)_2_	470 ± 70	IPFP	1370 ± 30

**Table 3:** Atomic polarizabilities (α, a.u.) of chalcogen (Ch) elements, van der Waals radii (R_vdW_, Å) and σ-hole MEP values (MEP, kcal/mol) of their difluoride derivatives taken from ref. [63].

Ch	α	R_vdW_	MEP (ChF_2_)
O	3.0	1.52	16.8
S	11.8	1.80	35.6
Se	17.5	1.90	44.9
Te	25.9	2.06	52.6
